# Supplementary material for: The genome sequence of the Poplar Cosmet moth, Batrachedra praeangusta (Haworth, 1828)
Source: Wellcome Open Res. 2025 Jun 2;10:288. [Version 1] doi: 10.12688/wellcomeopenres.24308.1 (PMC12395131; doi:10.12688/wellcomeopenres.24308.1)
Supplement: Peer review history

# Reviewer report: Reviewer response for version 1

**DOI:** 10.21956/wellcomeopenres.26808.r125487

**Published:** 2025-08-28

**Authors:** Arun Arumugaperumal

**Affiliations:** 1 Rajalakshmi Engineering College, Chennai, Tamil Nadu, India

The data note reports the genome sequence of the poplar cosmet moth *Batrachedra praeangusta*. It is advisable that the second author can also have ORCID linked to his name like what has been done with the first author name. The assembly reported here is of size 486.93 Mb. The mitogenome was also provided separately. The specimen collected was a female. I suppose this belongs to ZZ/ZO chromosome system. Under the heading 'Sequencing data' change '121 coverage' to '121x coverage'. The organism photograph was clearly recorded in figure 1. The accession IDs reported were working fine. The data note can be indexed.

Are sufficient details of methods and materials provided to allow replication by others?

Yes

Is the rationale for creating the dataset(s) clearly described?

Yes

Are the datasets clearly presented in a useable and accessible format?

Yes

Are the protocols appropriate and is the work technically sound?

Yes

Reviewer Expertise:

Bioinformatics; Genomics

I confirm that I have read this submission and believe that I have an appropriate level of expertise to confirm that it is of an acceptable scientific standard.

# Reviewer report: Reviewer response for version 1

**DOI:** 10.21956/wellcomeopenres.26808.r125489

**Published:** 2025-08-14

**Authors:** Sivasankaran Kuppusamy

**Affiliations:** 1 Loyola College, Chennai, Tamil Nadu, India

Authors have done the chromosome-level genome sequence of *Batrachedra praeangusta* (Haworth, 1828). They produced 201.52 Gb genome. They assembled and annotated using appropriate softwares. They detected 29 chromosomal pseudomolecules through the assembly.  

**Comments on the manuscript**

*Batrachedra praeangusta* (Haworth, 1828) Poplar Cosmet is a moth belonging to the family Batrachedridae. There are about 140 species in the genus *Batrachedra* worldwide. Among them, three are in the UK (Hall & Plant, 2022), with most species in the tropics.

 The genome of the *B. praeangusta* was sequenced as part of the Darwin Tree of Life Project…

 The author used the full name of the genus, *Batrachedra praeangusta*, throughout the manuscript. If it’s possible, begin with the full genus name and then abbreviate it to *B. praeangusta.*

**

 Authors have sequenced 201.52 Gb genome size using different sequencing platforms. They assembled and annotated the genome sequence. But they haven’t included the annotation list of the genomes like a number of protein-coding genes, non-coding genes and gene transcripts.

 The research article was well prepared and the manuscript meets the necessary scientific standard and is suitable for indexing

Are sufficient details of methods and materials provided to allow replication by others?

Yes

Is the rationale for creating the dataset(s) clearly described?

Yes

Are the datasets clearly presented in a useable and accessible format?

Yes

Are the protocols appropriate and is the work technically sound?

Yes

Reviewer Expertise:

Phylogenomics

I confirm that I have read this submission and believe that I have an appropriate level of expertise to confirm that it is of an acceptable scientific standard.
